# PSMA-PET based radiotherapy: a review of initial experiences, survey on current practice and future perspectives

**DOI:** 10.1186/s13014-018-1047-5

**Published:** 2018-05-11

**Authors:** Sebastian Zschaeck, Fabian Lohaus, Marcus Beck, Gregor Habl, Stephanie Kroeze, Constantinos Zamboglou, Stefan Alexander Koerber, Jürgen Debus, Tobias Hölscher, Peter Wust, Ute Ganswindt, Alexander D. J. Baur, Klaus Zöphel, Nikola Cihoric, Matthias Guckenberger, Stephanie E. Combs, Anca Ligia Grosu, Pirus Ghadjar, Claus Belka

**Affiliations:** 1 Department of Radiation Oncology, Klinik für Radioonkologie und Strahlentherapie, Charité Universitätsmedizin Berlin, Berlin, Germany; 2 Department of Radiation Oncology, Faculty of Medicine and University Hospital Carl Gustav Carus, Technische Universität Dresden, Dresden, Germany; 3 German Cancer Research Center (DKFZ), Heidelberg and German Cancer Consortium (DKTK), Dresden, Germany; 4 Department of Radiation Oncology, Technical University of Munich (TUM), Munich, Germany; 5 Institute of Innovative Radiotherapy (iRT), Department of Radiation Sciences (DRS), Helmholtz Zentrum München (HMGU), München, Germany; 6 Department of Radiation Oncology, University Hospital Zurich, Zurich, Switzerland; 7 Department of Radiation Oncology, Medical Center - University of Freiburg, Faculty of Medicine, University of Freiburg, Dresden, Germany; 8 German Cancer Consortium (DKTK), Partner Site Freiburg, Dresden, Germany; 9 Department of Radiation Oncology, University Hospital Heidelberg, Heidelberg, Germany; 10 German cancer research center (DKFZ) and german consortium for translational cancer research (DKTK), Heidelberg, Germany; 11 Department of Radiation Oncology, Ludwig-Maximilians-University, Munich, Germany; 12 German Cancer Research Center (DKFZ), Heidelberg and German Cancer Consortium (DKTK) partner site Munich, Munich, Germany; 13 Department of Therapeutic Radiology and Oncology, Innsbruck Medical University, Innsbruck, Austria; 14 Department of of Radiology, Universitätsmedizin Berlin, Berlin, Germany; 15 Nuclear Medicine Department, University Hospital Carl Gustav Carus, TU Dresden, Dresden, Germany; 16 Department of Radiation Oncology, Inselspital, Bern University Hospital, University of Bern, München, Switzerland; 17 Charité Universitätsmedizin Berlin, Klinik für Radioonkologie und Strahlentherapie, Augustenburger Platz 1, 13353 Berlin, Germany

**Keywords:** PSMA-PET, Prostate-cancer, Salvage radiotherapy, Primary radiotherapy, Image guided treatment planning, Review, Survey

## Abstract

^68^Gallium prostate specific membrane antigen (PSMA) ligand positron emission tomography (PET) is an increasingly used imaging modality in prostate cancer, especially in cases of tumor recurrence after curative intended therapy. Owed to the novelty of the PSMA-targeting tracers, clinical evidence on the value of PSMA-PET is moderate but rapidly increasing. State of the art imaging is pivotal for radiotherapy treatment planning as it may affect dose prescription, target delineation and use of concomitant therapy.

This review summarizes the evidence on PSMA-PET imaging from a radiation oncologist’s point of view. Additionally a short survey containing twelve examples of patients and 6 additional questions was performed in seven mayor academic centers with experience in PSMA ligand imaging and the findings are reported here.

**Electronic supplementary material:**

The online version of this article (10.1186/s13014-018-1047-5) contains supplementary material, which is available to authorized users.

## Background

**Fig. 1:**
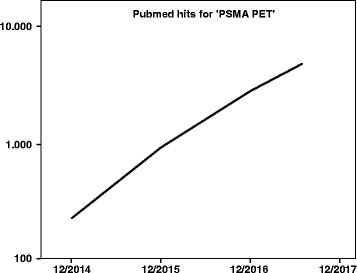
Number of results for the search term “psma pet” in pubmed.gov with annual publication date restrictions starting from January 2014 until July 2017 (x-axis) and logarithmic depiction of cumulative publications (y-axis)

Positron emission tomography (PET) imaging with ^68^Gallium-labeled prostate specific membrane antigen ligands (PSMA) for prostate cancer patients has entered clinical practice for staging prior to radiotherapeutic treatment, especially for high-risk tumors and patients suffering biochemical recurrence after surgery. As PET is usually performed in combination with computed tomography (CT) for attenuation correction and anatomical information, the term PSMA PET is subsequently used as an abbreviation for this combined examination, unless otherwise stated. PSMA-PET has a higher specificity and sensitivity for the detection of tumor lesions compared to stand alone CT, magnetic resonance imaging (MRI) and Choline-PET. It offers promising opportunities for treatment individualization [1, 2]. PSMA-PET (CT/MRI) was introduced in 2012 [3–5]. Its clinical use and the scientific interest in PSMA-PET imaging increased almost exponentially as suggested by a Pubmed search using the terms PSMA PET (Fig. 1). Due to the relative novelty of this radiotracer there is a steadily increasing clinical evidence for the implementation of PSMA PET for clinical decision making and radiotherapeutic target volume delineation. Despite sparse high-grade evidence, it was shown that PSMA-PET imaging had influence on radiotherapy treatment in more than 48% of high risk patients (treatment-naïve and recurrent prostate cancer) [6]. In two recent publications with 161 and 270 patients suffering biochemical recurrence Calais and colleagues reported intended treatment management changes in more than 50% of patients. In case of early biochemical recurrence (defined as PSA < 1.0 ng/ml) there was still a major impact of further treatment planning in 19% of patients [7, 8].

This review focuses on the use of PSMA-PET for radiotherapy planning and treatment, based on the clinically most widely used ^68^Gallium-labeled PSMA ligands. The probably most important issues regarding PSMA-PET for radiation oncologist are: When to use PSMA-PET imaging for treatment planning/ staging and how to optimally adapt radiotherapy planning to PSMA-PET findings. The limitations and caveats of ^68^Gallium-PSMA ligand and methods to improve imaging quality and detection rates by alternative acquisition protocols or PSMA conjugates are only briefly mentioned as they were already comprehensively discussed elsewhere [9–12].

### PSMA-PET for primary staging and definitive radiotherapy

The role of PSMA-PET for primary staging of prostate cancer is less well defined as the potential important role for biochemical recurrence after treatment with curative intent. In a prospective multicenter assessment on treatment modification by PSMA-PET in 108 treatment-naïve intermediate and high-risk patients, PSMA-PET led to treatment modifications in 21% [13]. Surprisingly, there was no statistical significant difference of treatment alterations between intermediate and high-risk patients in this largest published prospective cohort of primary staged patients. Dewes and colleagues reported on 15 patients treated with definitive radiotherapy. The radiotherapeutic concept was changed in 33% of patients due to the PSMA-PET CT, mostly leading to additional target volumes/ dose escalation due to detected pelvic lymph node metastases [14].

In a meta-analysis on the role of PSMA-PET for primary staging of prostate cancer, von Eyben and colleagues identified seven studies, mostly retrospective analyses of consecutive patients. PSMA-expressing lesions were only identified in 203 of 273 patients (74%): 60% had lesions confined to the prostate, 4% pelvic lymph nodes and 10% presented lesions in more than one site, i.e. treatment was probably adapted in around 14% of patients [2]. This intriguingly low number of PSMA expression within the primary tumor lesions requires a closer look at the mentioned publications. Some of the seven reviewed studies apparently only received dissection of pelvic lymph nodes with unknown treatment status of the primary tumor: Budäus et al. focused on lymph node detection rates and didn’t explicitly report on primary tumors, additionally even patients with prostate specific antigen (PSA) values as low as 1.4 ng/ml were included [15]. Similarly, the analyses of Herlemann et al. and van Leeuwen et al. were restricted to lymph node detection [16, 17]. Fendler et al. reported a detection rate of intraprostatic PMSA lesions of 90% (19 of 21 patients) [18]. Maurer et al. reported a positive detection rate within the prostate of 91.6% in an analysis of 130 patients [19]. Rhee et al. reported lesion based analyses only [20]. The probably most interesting study from a radiation oncologists perspective from Zamboglou et al. reported PSMA-PET based delineation. In 22 of 23 patients a gross tumor volume could be generated by the use of PSMA-PET: Only in one patient no target volume could be delineated based on PSMA PET. This means 95% of patients would have had potentially suspicious PSMA uptake within the prostate [21].

In summary these data show that the detection rate of intraprostatic PSMA-positive lesions should be up to 95% (when primary lesions are separately analyzed as discussed above). Nevertheless, sensitivity for detection of every intraprostatic cancer focus (i.e. pathological confirmed tumor localization) remains relatively low with a pooled sensitivity around 70% and a specificity around 84% [2]. Rhee, Eiber, Zamboglou and colleagues compared multiparametric MRI, PSMA-PET and detection of histologic lesions: PSMA-PET outweighed MRI, but in a relevant number of cases MRI and PSMA-PET delivered complementary information on localization of lesions [20, 22, 23]. Since PSMA uptake correlated with features of tumor aggressiveness like extracapsular infiltration or Gleason score, a miss of small or lower grade intraprostatic lesions may be acceptable for radiotherapy treatment planning [18], as PET volumes would currently only be used to deliver a boost dose within the prostate. Poorly differentiated prostate cancer cells are known to be more radioresistant than well differentiated ones, hence higher Gleason scores were associated with markers of radioresistance and increased rates of local recurrences after primary radiotherapy in some but not all studies [24–27]. Therefore, higher radiation doses (e.g. by a PET based boost) are potentially only needed for tumors showing aggressive features while the lower (standard) dose to the surrounding prostate should be sufficient to treat small or low grade lesions.

The concept of biological guided dose escalation (i.e. prescription of radiation dose according to voxelwise PET tracer uptake) was first propagated more than 10 years ago [28]. Most studies on PET guided dose-painting focused on ^18^F-fluorodesoxyglucose (FDG) or hypoxia PET tracers. However, many concerns have been raised about the utility of the latter one for dose-painting, as the lesion to background ratio is usually low and stability of the tracer distribution within the tumor doubtful. This may be a reason why no practice changing studies on dose-painting approaches have been published so far. PSMA ligands seem to be promising tracers for radiotherapeutic dose-escalation as the correlation with histopathological findings is relatively high [18, 23, 29]. Dose-escalation for definitive treatment of prostate cancer has shown to provide relevant improvement of progression free survival, albeit at the cost of higher early and potentially more pronounced late toxicities [30, 31]. In a study by Budäus and colleagues intraprostatic foci were correctly predicted by PSMA-PET imaging before radical prostatectomy in 93% of patients in an analysis of 30 patients [15]. Semi-automatic PSMA contouring with an intraprostatic threshold of 30% of the intraprostatic SUV_max_, used for gross tumor delineation, was proven to be technically feasible and would be relatively easy reproducible e.g. within a multi-center trial [32]. Additionally, the level of PSMA ligand uptake correlated with established risk factors like Gleason or d’Amico risk groups [33] and histological studies proved that cellular PSMA expression and PET uptake correlated with features of tumor aggressiveness [34]. Multi-parametric dose-painting for prostate cancer using MRI and PET information is technically feasible; however, MRI and PET often show a relatively large overlap. Furthermore, it remains unknown if the additional information of MRI and PET imaging adds information for treatment planning which would be clinically meaningful [35, 36]. A planning study on PSMA-PET based dose-escalation within sub-volumes of the prostate by Zamboglou and colleagues showed a promising increase of tumor control probability, without negatively affecting normal tissue complication probabilities in modeled patients [32].

Although PSMA-PET has higher detection rates of lymph node metastases compared to conventional imaging or Choline-PET, due to the inherent limitation of PET imaging, microscopic spread or affected small volume lymph nodes are potentially missed by PET imaging. Pooled comparison with surgical specimens revealed a high specificity of 97%, but a moderate sensitivity of around 61% [2]. However, sensitivity is improved compared to CT, MRI or Choline-PET imaging [37–41]. One study reported a median diameter of false-negative lymph nodes (i.e. PSMA-PET negative but histopathological positive) of only 1.3 mm [42]. However, a negative finding in PSMA-PET is not able to rule out metastatic spread within tiny lymph nodes.

For the detection of distant (bone) metastases, PSMA-PET has a higher detection rate than standard bone scintigraphy regarding lesion number [43]. Additional bone scans seem to be dispensable if a PSMA-PET was performed [44].

### PSMA-PET for PSA persistence or biochemical recurrence after radical prostatectomy

Salvage radiotherapy for biochemical recurrent disease should be performed as early as possible [45–47]. However, detection rates for PSMA-PET depend highly on PSA levels. While salvage radiotherapy should optimally be initiated with PSA levels < 0.5 ng/ml [47], the rate of PSMA positive tumor manifestation is relatively low in this PSA range. Afshar-Oromieh and collegues reported on 1007 consecutive patients and found PSMA-positive lesions in 48% for PSA values ≤0.5 ng/ml and 73% for PSA values between 0.5 ng/ml and 1.0 ng/ml [48], similarly Eiber and colleagues reported a positive detection rate of 57.9% [49]. Rauscher and colleagues recently published data of 272 patients with early biochemical recurrence. PSMA positive lesions were evident in 55% of patients with PSA values between 0.2 and 0.5 ng/ml and 74% of patients with PSA values between 0.5 and 1.0 ng/ml [50]. Other studies with smaller sample sizes reported a positive detection rate of 44% for PSA values < 1 ng/ml and 48% for PSA values < 0.8 ng/ml [51, 52]. Therefore, based on the largest series of patients from Afshar-Oromieh and collegues, PSMA-PET should probably be performed in case of PSA levels > 0.5 ng/ml due to the relatively high detection rates of 70% or more. However, in some cases PSMA-PET detects lesions even in patients with very low PSA values: An analysis of 70 patients reported relatively high detection rates of 58% even for PSA values between 0.20–0.29 ng/ml [53]. A limitation of these studies is that patients receiving salvage radiotherapy for biochemical recurrence were not separately analyzed from patients receiving salvage radiotherapy for postoperative persisting PSA levels. Additionally, detailed information on concomitant ADT use was often missing. In cases of very high risk situations for regional or distant spread, e.g. R0 resection and persisting PSA values, PSMA-PET might be performed even with PSA values < 0.5 ng/ml. This pre-selection of patients with low risk for isolated local recurrence may be useful since detection of small lesions around the former prostate gland can be difficult due to the high urinary bladder spillover [9]. This constraint can only partially be resolved by images at later time points (tracer dilution).

PSMA-PET leads to radiotherapy treatment modification in up to 59% of cases presenting with biochemical recurrence at the radiation oncology department, as reported in a recent publication that included 100 patients with a median PSA level of 1.0 ng/ml [54]. Similar rates of radiotherapy adaptation were found in other publications with smaller sample sizes [6, 41, 55]. Interestingly, PSMA-positive lymph nodes would not be covered by delineation of the pelvic lymphatic drainage according to RTOG lymph node target volume recommendations in up to 40% of cases [56, 57]. The ever increasing experience with PSMA-PET imaging may on the long run lead to alterations of the recommended pelvic target volume delineation.

A current Australian study reported a cohort of 164 patients referred for salvage radiotherapy. PSA levels were between 0.05 and 1 ng/ml and PSMA positive lesions were detected in 61%. PSA response after salvage radiotherapy in patients not receiving androgen deprivation therapy was highest in case of missing evidence of PET lesions or disease limited to the prostate fossa, with 86 and 83% respectively. Nodal involvement reduced response rate to 62% (after nodal irradiation) and distant metastases further reduced post-radiotherapeutic response to 30% [58]. However, this may only be true in case of relatively low PSA values up to 1 ng/ml, as another publication that included patients with higher PSA values reported a potential unfavourable PSA response after radiotherapy limited to the prostatic region in case of negative PSMA findings for these patients [59].

Table 1 summarizes the current literature on biochemical response after PSMA guided radiotherapy. Although low patient numbers and a short follow-up are common limitations to all studies, some cautious conclusions may already be drawn: For low PSA values, salvage radiotherapy of the prostate fossa should not be omitted in case of PSMA-negativity. The probability of lasting PSA response after radiotherapy is probably highest for local recurrence, intermediate for regional and distant lymph node recurrences and lowest for bone metastases. Nevertheless, some patients with distant metastases seem to present an intermediate-term PSA response. At the moment, it is not predictable which patients with distant metastases benefit most likely from high-dose PSMA based radiotherapy.

**Table 1:** First reports on biochemical response following PSMA based radiotherapy after prior radical treatment of the prostate

Referal for salvage/ non primary radiotherapy, PSA response								
Author	Number of irradiated patients	Treatment of	Median PSA value	ADT use before radiotherapy	Follow up time (months)	PSA response during follow up	Re-Radiation	ADT initiation during follow up
Henkenberens [69]	23	N, Ma,	2.75	0%	12.4	52%	26%	22%
Zschaeck [59]	20 (11)	T, N, Ma, Mb	0.95	45% (0%)	29.0	70% (73%)	15% (27%)	5% (9%)
Bluemel [70]	43	T, N, Ma, Mb	0.60	0%	8.1	83%^a^	0%	n.a.
Emmett [58]	99	T, N, Ma, Mb	0.28	0%	10.5	72%	n.a.	n.a.
Habl [61]	15	Mb	1.99	20%	22.5	20%	7%	n.a.
Henkenberens [71]	29	T, N, Ma, Mb	1.47	38%	8.3	96%	7%	0%
Guler [68]	23	N, Ma, Mb	1.10	100% (43%^b^)	7.0	83%	0%	9%

T = local recurrence, N = pelvic lymph nodes, Ma = extrapelvic lymph nodes, Mb = bony metastases. *ADT* androgen deprivation therapy

^a^Follow up only available for 21 patients: 20 presenting good response, however 3 patients with rising PSA values during salvage irradiation were included to the non-responders (4/24 patients)

^b^43% of patients presented with hormone refractory situation after long-term ADT

### PSMA-PET for the treatment of (oligo-)metastatic prostate cancer

The concept of oligometastases was introduced by Hellman and Weichselbaum in 1995. They stated that “as effective chemotherapy becomes more widely applicable, there should be another group of patients with oligometastases. These are patients who had widespread metastases that were mostly eradicated by systemic agents, the chemotherapy having failed to destroy those remaining because of the number of tumor cells, the presence of drug-resistant cells, or the tumor foci being located in some pharmacologically privileged site” [60].

Due to the high detection rate of prostate cancer manifestations, PSMA-PET seems to be highly promising for detection and treatment of oligometastatic disease. First retrospective data on the treatment of oligometastases was published by Habl et al.: They analyzed 15 patients with a total of 20 bone metastases who underwent high-dose stereotactic radiotherapy and reported a median biochemical progression free survival of 6.9 months [61]. Several prospective studies on the role of radiotherapy to oligometastatic lesions are currently recruiting [62].

PSMA based irradiation of gross PSMA-positive tumor lesions may even have a potential to restore hormone-response: PSMA expression is upregulated after androgen deprivation therapy (ADT) and higher expressed in biological more aggressive tumors [63]. Selective pressure on tumor cells by ADT may lead to (oligo-) progressive high PSMA expressing disease [64]. A recent publication reported one case of restored hormone-response after radioligand therapy [65]. PSMA based irradiation of bulky disease may be a promising approach in oligo-recurrent/ progressive disease after ADT. Pre-clinical studies and some clinical data reported enhanced cellular PSMA expression after ADT and a potential association of castration-resistant status and increase of PSMA expression. Additionally, maximal PSMA standardized uptake values (SUV_max_) of hormone refractory oligometastatic patients were higher than these of castration sensitive patients [63, 66–68]. A recent publication describing 23 patients treated for evidence of oligometastatic prostate cancer on PSMA-PET included 10 castration resistant patients with Median PSA values of Median 5.5 ng/ml [68]. In this poor prognosis group the authors reported a median progression free survival of 7 months. However, it remains unclear how progression was classified. A closer look at the patients revealed that 9 out of 10 patients had lower PSA values during follow-up compared to the value before radiotherapy. Five of them presented PSA decreases > 50% at the last follow up visit. In this small group this rate was quite favorable when compared to PSA decreases in only 8 out of 13 hormone-sensitive patients.

### Current practice

We performed a short survey containing twelve short example cases depicting typical clinical scenarios and 6 additional questions (opinion on the value of PSMA-PET, effect of PSMA-PET on treatment) in seven mayor university centers with experience in PSMA-PET imaging. Based on clinical parameters and findings of PSMA-PET we asked for an institutional consensus to suggest treatment recommendations but accepted alternative answers if no consensus was achievable. Twelve questionnaires were included for evaluation. All participants were experienced radiation oncologists with a median time of 10 years in practice and 3 years of experience with PSMA based radiotherapy. Since case reports were relatively short many radiation oncologists demanded additional MRI imaging or pathological information. The detailed information on the patient cases and the respective answers can be found in Additional file 1. When asked for the influence of PSMA-PET on radiotherapeutic management, it was considered to alter treatment in 60% of cases (median). In case of PSA persistence or recurrence without evidence of PSMA-positive lesions, the large majority of radiation oncologists would opt for salvage radiation of the prostate fossa (100% in case of PSA recurrence of 0.26 ng/ml and 92% in case of PSA persistence or PSA recurrence with a PSA value of 2.9 ng/ml). Interestingly, in the latter case 83% would recommend additional androgen deprivation therapy, while only 18% would recommend additional irradiation of the lymphatic drainage.

Remarkably, a very high unanimity existed in case of PSMA evidence of positive pelvic nodes: 92–100% would recommend radiation of prostate fossa, pelvic lymphatic drainage and boost of PSMA positive lesions, with additional androgen deprivation therapy being recommended by most centers irrespective of surgical resection status. In case of two para-aortic lymph node metastases in a R1 resected patient with a Gleason score of 7 an pN+, PSA persistence and a PSA value before radiotherapy of 1.6 ng/ml, most radiation oncologists would recommend ADT (92%) with one limiting the treatment to ADT only. The majority would additional irradiate pelvic lymphatic drainage (75%) and para-aortic nodes (83%), while only 58% would recommend to additionally irradiate the prostate fossa. Only 18% would (alternatively) offer stereotactic ablative radiotherapy of the two lymph nodes. In case of the same para-aortic findings, but in a heavily pre-treated patient (surgery, radiotherapy to the fossa, ADT and hormone-refractory situation with PSA of 0.72 ng/ml) most radiation oncologist would opt for intensification of systemic treatment (18% would only recommend this treatment without any radiotherapy), but still 83% would irradiate PSMA-PET findings either as a boost to para-aortal nodes (67%) or by stereotactic radiotherapy (18%). If the same patient would present without para-aortal but with three lymph nodes restricted to the pelvis 92% would recommend irradiation of pelvic lymphatic drainage with a boost to PSMA positive lesions and 75% (additional) systemic treatment intensification.

In case of a patient with Gleason score of 8 and R0 with pre-radiotherapeutic PSA level of 2.1 ng/ml and a solitary bone metastasis, 91% would irradiate the lesion (stereotactically or fractionated), mostly also recommending systemic treatment/ ADT (64%), only 9% would not irradiate an asymptomatic bone lesion and no one would irradiate prostatic fossa or pelvic nodes. This would however dramatically change if the same patient would present two additional pelvic lymph nodes: 91% would irradiate the lymphatic drainage with a boost to PSMA positive lesions and 75% would also irradiate the prostatic fossa. When the same respondents were asked about their tendency not to irradiate prostate fossa (1 = would definitely not irradiate up to 9 = would definitely irradiate) in a patient with high-risk features who presented postoperative PSA persistence, this was heavily influenced by PSMA-PET findings. In case of lymphatic nodes restricted to the pelvis the majority would irradiate the prostate fossa (Average value: 8.3), however, if only extrapelvic lymph node metastases were evident many radiation oncologists tended not to irradiate (Average 4.1).

In case of the patient described above with castration resistance and two bone lesions detected by PET, 83% would opt for intensification of systemic treatment and 67% would offer additional high-dose irradiation of these lesions.

**Fig. 2:**
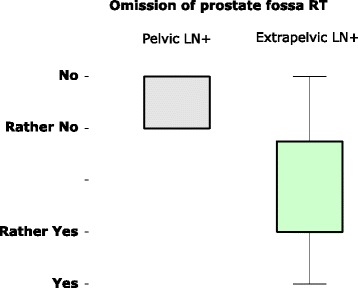
Willingness of twelve radiation oncologists to omit radiotherapy to the prostate fossa (prostate fossa RT). Asked if they would consider omission of prostate fossa RT in a high-risk patient after completely resected (R0) prostate cancer with persisting PSA values (> 0.6 ng/ml). Possible disposition had to be scored and ranged from 1 (definitely yes) to 9 (definitely no). Most radiation oncologists were in favour of prostate fossa RT in case of PSMA positive lesions within the pelvis (left), a high disagreement existed in case of extrapelvic PSMA positive lymph node lesions as only finding in PET (right). Boxplot showing 50% quartiles, whiskers showing whole range of given responses

Most dissent related to omission of radiotherapy of the prostate fossa in case of extrapelvic PSMA positive lesions in a high risk patient with completely resected prostate cancer and persisting postoperative PSA. Figure 2 depicts the willingness to omit radiotherapy to the prostate fossa depending on PSMA-PET findings.

## Conclusion

PSMA-PET should be considered the actual gold standard for imaging of biochemical recurrent prostate cancer, outperforming conventional imaging and Choline-PET in regard to sensitivity and specificity for detection of lymph node and distant metastases. PSMA-PET imaging should be recommended for PSA values > 0.5 ng/ml after radical prostatectomy. For biochemical recurrence with PSA values < 0.5 ng/ml or treatment-naïve intermediate or high-risk patients there is no good evidence if and when to use PSMA-PET imaging for staging. Therefore, additional clinical risk factors and potential therapeutic consequences should be carefully considered for each patient. Current clinical guidelines like the German S3 guideline underline the importance of PSMA-PET imaging for recurrent prostate cancer, even as upfront diagnostic approach, while recommending its use in treatment-naïve prostate cancer patient staging within prospective trials only [47]. Initial studies with limited numbers of patients and short follow-up time showed promising biochemical responses in the majority of patients that were treated for PSMA-positive recurrent tumor lesions. However, it is impossible to know yet if this affects overall survival. By our short survey we identified the most critical radiation oncology issues: When to irradiate prostate fossa in case of loco-regional or distant PET findings and how extensive radiation fields should be in case of localized extrapelvic lymph node metastasis? Additionally, there is no published data on potential synergies of ADT and PSMA based external beam radiotherapy as well as PSMA based radioreceptor therapy. This should be further elucidated by pre-clinical models and prospective clinical trials.

## Additional file

Additional file 1: Detailed information on individual cases and the respective answers of all participating radiation oncologists. (DOCX 93 kb)

## Abbreviations

ADT: Androgen deprivation therapy
CT: Computed tomography
FDG: 18F-fluorodesoxyglucose
Gy: Gray
iPSA: Initial PSA value
ml: milliliter
MRI: Magnetic resonance imaging
ng: Nanogram
PET: Positron emission tomography
PSA: Prostate specific antigen
PSMA: Prostate specific membrane antigen
RT LD: Radiotherapy to the pelvic lymphatic drainage
RT PA-LD: Radiotherapy to the para-aortic lymph nodes
RT Prostate Fossa: Radiotherapy to the prostate fossa
RTOG: Radiation Therapy Oncology Group
SRT: Stereotactic radiotherapy
SUV_max_: Maximal standardized uptake value
