# Supplementary material for: PSMA-PET based radiotherapy: a review of initial experiences, survey on current practice and future perspectives
Source: Radiat Oncol. 2018 May 11;13:90. doi: 10.1186/s13014-018-1047-5 (PMC5948793; doi:10.1186/s13014-018-1047-5)

**Additional file 1**

**Case 1 (n=11):**

iPSA 8.4 ng/ml, Gleason score 7, pN0, R1, PSA Nadir: 0.05 ng/ml. Three consecutively rising PSA values up to 0.26 ng/ml.

PSMA-PET-CT: No detectable lesions

Irradiation of prostate fossa: 100%

**Case 2 (n=12):**

Gleason 7, pN0, R0, persisting PSA value after surgery, actual PSA value 0.58 ng/ml.

PSMA-PET-CT: No detectable lesions


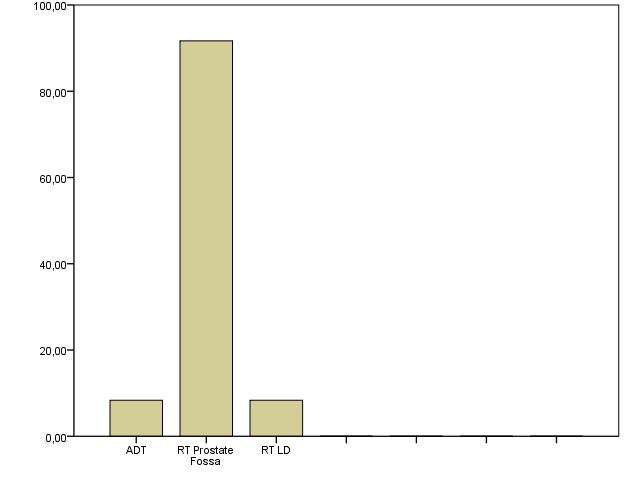


**Case 3 (n=12):**

iPSA 8.4 ng/ml, Gleason score 7, pN0, R0, PSA Nadir: 0.14 ng/ml. Consultation at Radiotherapy department with PSA of 2.9 ng/ml

PSMA-PET-CT: No detectable lesions


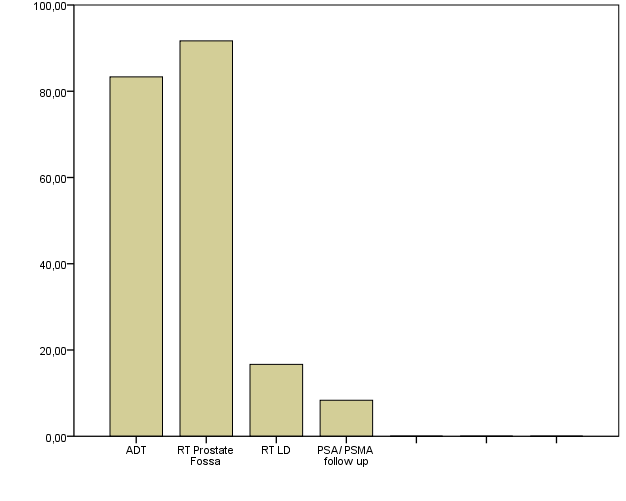


**Case 4 (n=12)**

iPSA 6.4 ng/ml, Gleason score 7, pN0, R1, PSA Nadir: 0.04 ng/ml. Consultation at Radiotherapy department with PSA of 0.8 ng/ml

PSMA-PET-CT: Local recurrence, cN0, cM0


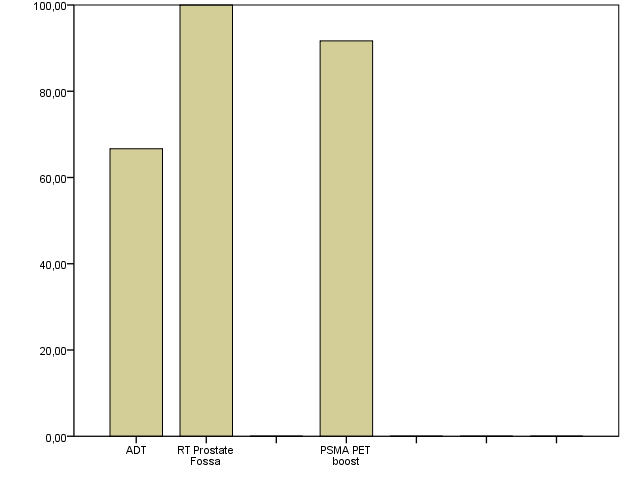


**Case 5 (n=12)**

iPSA 12.7 ng/ml, Gleason score 8, pN0, R0, PSA Nadir: 0.12 ng/ml. Consultation at Radiotherapy department with PSA of 0.8 ng/ml

PSMA-PET-CT: Two pelvic lymphnode metastases, cM0


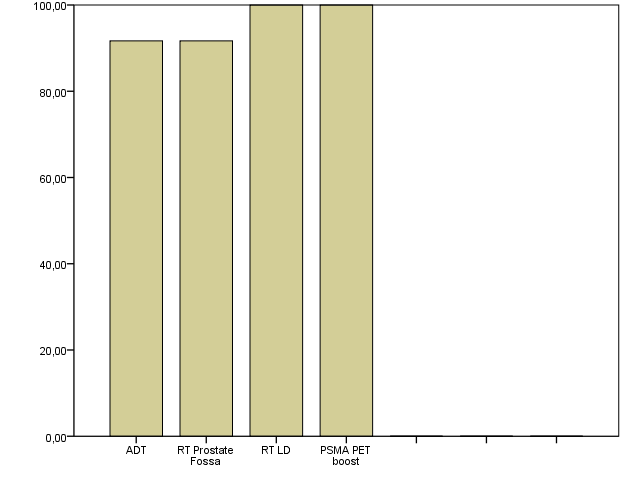


**Case 6 (n=12)**

iPSA 12.7 ng/ml, Gleason score 8, pN0, R1, PSA Nadir: 0.12 ng/ml. Consultation at Radiotherapy department with PSA of 0.8 ng/ml

PSMA-PET-CT: Two pelvic lymphnode metastases, cM0


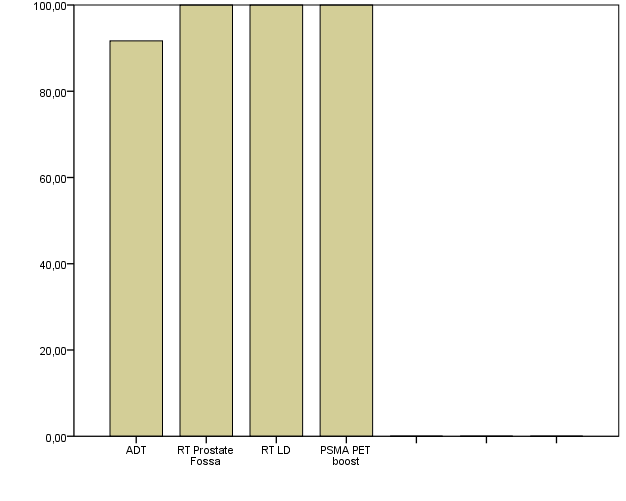


**Case 7 (n=11)**

iPSA 16.0 ng/ml, Gleason score 8, pN0, R0, PSA Nadir: 0.41 ng/ml. Consultation at Radiotherapy department with PSA of 2.1 ng/ml

PSMA-PET-CT: Solitary bone lesion


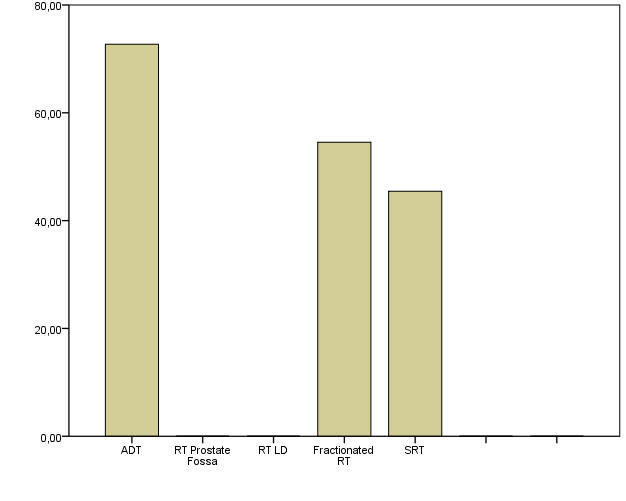


**Case 8 (n=11)**

iPSA 16.0 ng/ml, Gleason score 8, pN0, R0, PSA Nadir: 0.41 ng/ml. Consultation at Radiotherapy department with PSA of 2.1 ng/ml

PSMA-PET-CT: Bone lesion + additional two pelvic lymphnode metastases


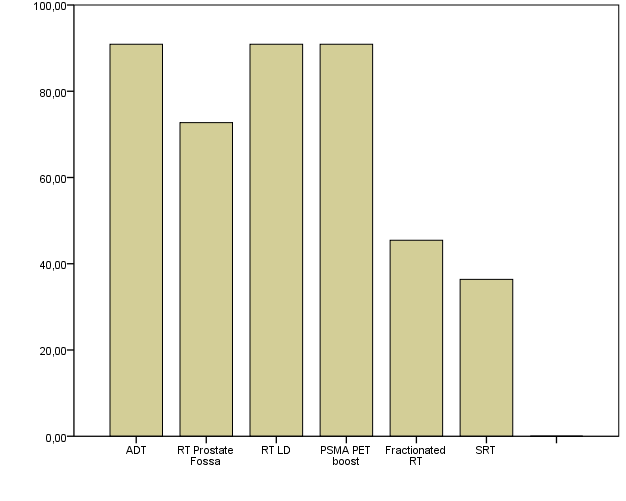


**Case 9 (n=12)**

iPSA 12.5 ng/ml, Gleason score 7, pN1, R1, PSA Nadir: 0.83 ng/ml. Consultation at Radiotherapy department with PSA of 1.6 ng/ml

PSMA-PET-CT: Two para-aortal lymphnode metastases


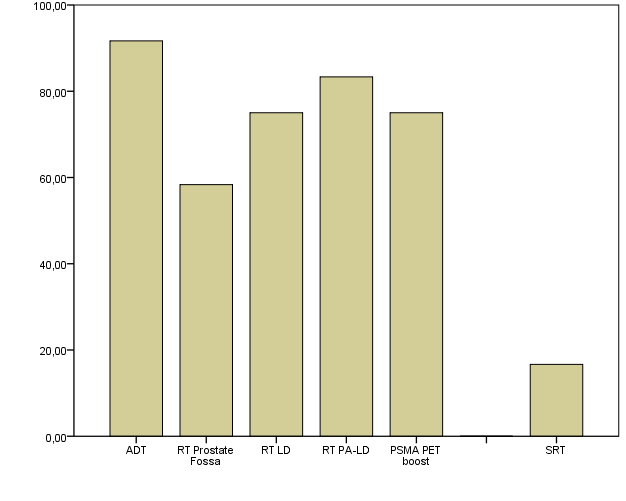


**Case 10 (n=12)**

Patient wit prior surgery and irradiation of prostate fossa (without LD), initiation of ADT after post-RT rising PSA values. Consultation at Radiotherapy department with rising PSA after > 3 years ADT, actual PSA value: 0.72 ng/ml

PSMA-PET-CT: Two para-aortal lymphnode metastases


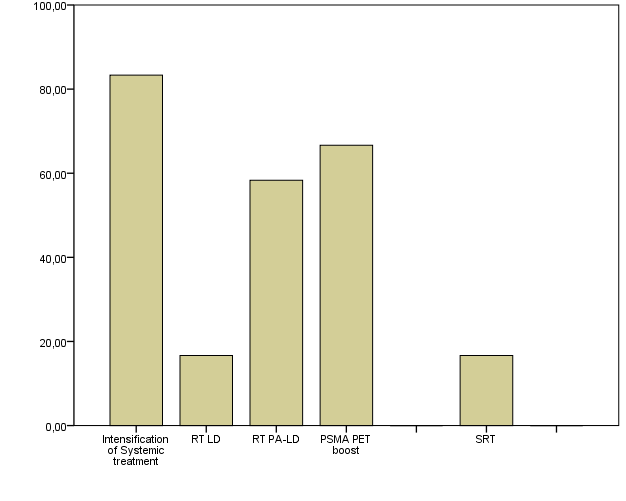


**Case 11 (n=12)**

Patient wit prior surgery and irradiation of prostate fossa (without LD), initiation of ADT after post-RT rising PSA values. Consultation at Radiotherapy department with rising PSA after > 3 years ADT, actual PSA value: 0.72 ng/ml

PSMA-PET-CT: Two bone metastases


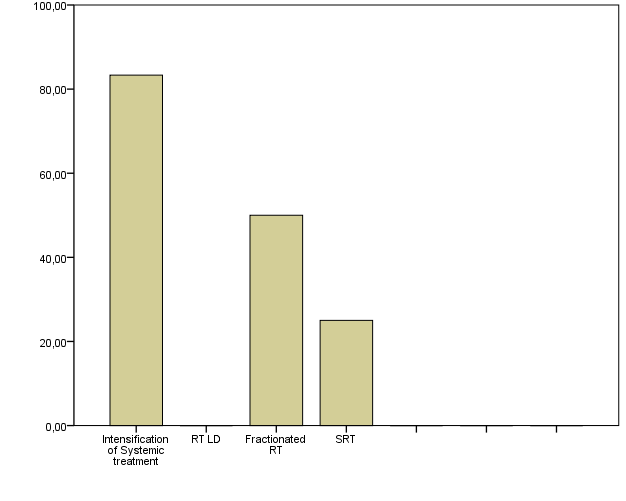


**Case 12 (n=12)**

Patient wit prior surgery and irradiation of prostate fossa (without LD), initiation of ADT after post-RT rising PSA values. Consultation at Radiotherapy department with rising PSA after > 3 years ADT, actual PSA value: 0.72 ng/ml

PSMA-PET-CT: three pelvic lyhmnode metastases


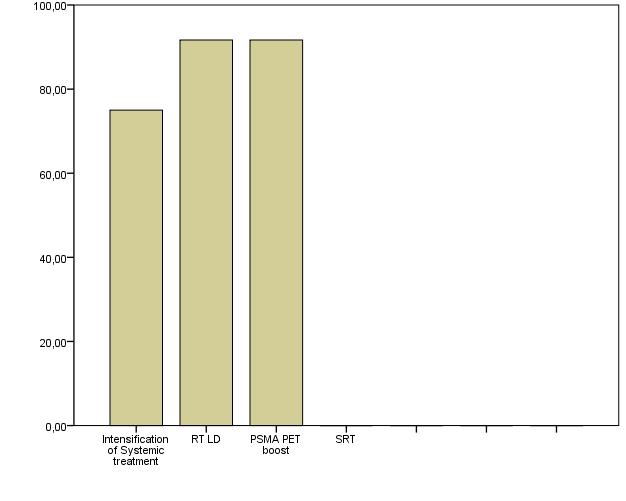

Supplement: Supplementary file 1 — Detailed information on individual cases and the respective answers of all participating radiation oncologists. (DOCX 93 kb) [file 13014_2018_1047_MOESM1_ESM.docx]
